# Fabrication and Characterization of a Microscale Piezoelectric Vibrator Based on Electrohydrodynamic Jet Printed PZT Thick Film

**DOI:** 10.3390/mi12050524

**Published:** 2021-05-06

**Authors:** Dazhi Wang, Kuipeng Zhao, Yuheng Yuan, Zhu Wang, Haoran Zong, Xi Zhang, Junsheng Liang

**Affiliations:** 1 Key Laboratory for Micro/Nano Technology and System of Liaoning Province, Dalian University of Technology, Dalian 116024, China; kuipengzhao@mail.dlut.edu.cn (K.Z.); Eternity666@mail.dlut.edu.cn (Y.Y.); studentwz@mail.dlut.edu.cn (Z.W.); 316059124@mail.dlut.edu.cn (H.Z.); zhangxi@dlut.edu.cn (X.Z.); jsliang@dlut.edu.cn (J.L.); 2 Ningbo Institute of Dalian University of Technology, Ningbo 315000, China; 3 Key Laboratory for Precision and Non-traditional Machining Technology of Ministry of Education, Dalian University of Technology, Dalian 116024, China

**Keywords:** micro piezoelectric vibrator, electrohydrodynamic jet, PZT thick film, simulation, rotational speed

## Abstract

This paper proposes a novel way of preparing a PZT thick film micro vibrator using the electrohydrodynamic jet (E-Jet) printing technique. Initially, a micro piezoelectric vibrator was simulated and designed for obtaining optimized structure, which has a total thickness of less than 600 µm. Subsequently, the PZT thick film element was directly printed on the elastic body using the E-Jet printing. This method avoids the glue fabrication process involved in the bulk piezoelectric fabrication, thus avoiding the limits of voltage drops, isolating and absorbing amplitude usually occurred in the vibrator having glue interface. It was observed that B02 and B03 modes were generated at frequencies of 29.74 and 79.14 kHz, respectively, and the amplitudes of B02 and B03 modes were 406 and 176 nm, respectively. The error between the simulation and test result in the B03 modal is only 0.35%, which indicates the accuracy of the simulation analysis and the fabrication process. The PZT thick film traveling-wave micro vibrator successfully realized bidirectional rotation of a rotor, with a maximum speed of 681 rpm, which also shows a linear relationship between excitation voltage and rotary speed. This paper provides an effective method for preparing a micro piezoelectric vibrator for MEMS ultrasonic devices, which simplifies the manufacturing process and enhances the performance of the piezoelectric vibrator.

## 1. Introduction

A piezoelectric vibrator is an important piezoelectric component that is capable of converting electrical energy into mechanical energy. Generally, piezoelectric vibrators consist of piezoelectric ceramics and metal bases, utilizing the inverse piezoelectric effect of piezoelectric ceramics to generate stretching vibrations and convert them into different mode vibrations by interacting with the ceramics and metal bases [1]. The micro vibrational deformation of piezoelectric ceramics is further amplified by the special structure of metal bases in order to realize the driving function. In particular, when the frequency of the external electric field is consistent with the natural frequency of the piezoelectric vibrator, the piezoelectric vibrator is in a state of mechanical resonance with the largest vibration amplitude, realizing the maximum conversion of electrical energy and mechanical energy. The rotary traveling wave piezoelectric vibrator has advantages in that it is comprised of a large area, has low friction losses, and possesses a high working efficiency, which has often been adopted as the stator of rotatory type ultrasonic motors [2,3] as well as the machining tool for surface ultrasonic polishing.

Manufacturing piezoelectric vibrators is usually combined with piezoelectric bulk and an elastic body using an epoxy adhesive. However, the bonding process of epoxy adhesive is very complex and causes the thermal depolarization of piezoelectric material to reduce the performance of the vibrator. Liu et al. [4] and Lu et al. [5] fabricated piezoelectric vibrators as drive sources for ultrasonic motors using the traditional method of bonding piezoelectric bulk and an elastic body with organic adhesives. In this regard, the bonding process of piezoelectric bulk vibrators requires complicated processes such as coating glue uniformly and aligning positions to paste the piezoelectric bulk. Moreover, the entire handling process requires a special fixture to exert pressure in the heating environment, which can harm the performance of the piezoelectric vibrator. Remarkably, it is difficult to control the coating thickness and uniformity of organic glue during the bonding process, which may easily form bubbles that can generate larger concentrated stress, resulting in performance instability and limiting the service life of the ultrasonic vibrator [6]. Specifically, micro scale ring-shape traveling wave vibrators need higher preparation requirements, which place high-consistency demands on the shape, size, electrical properties, and relative position of a few hundred microns of piezoelectric ceramics in each zone. Hence, it is not practical to use epoxy adhesives as it is difficult to ensure accurate alignment and consistency during preparation with regard to micro scale piezoelectric vibrators.

Additionally, the glue layer between the piezoelectric bulk and metal bases will weaken the conductivity and excitation field for piezoelectric vibrators while partially absorbing the vibration micro-amplitude due to the elasticity of the adhesive layer, resulting in a reduced vibrator amplitude generated by the piezoelectric materials [7]. Moreover, the output power changes since the epoxy mechanical properties change with temperature [8]. The Q_m_ value of the PZT ceramic of vibrators in an ultrasonic motor becomes smaller due to the adhesive layer [9,10], indicating that piezoelectric devices have more energy consumption during the ultrasonic vibration process [11]. In view of the simulation analysis of piezoelectric vibrators, due to the glue layer, some simulation results such as losses, resonance frequency, and vibrational amplitude have large errors compared to the measurement results [12,13,14], since it is difficult to set an accurate and consistent physical parameter with a true state for the glue layer during the simulation. 

PZT thin film was combined with MEMS processes, where no adhesive process is involved. However, the fabrication techniques involved photolithography, etching, sol-gel [15], and sputtering [16], representing a complex and time-consuming manufacturing process. Furthermore, the thickness of the thin films was limited in the range of a few hundred nanometers to several micrometers. Generally, a PZT thin film at such scales will be affected by size and interface effects [17,18], limiting the vibrational performances of thin film piezoelectric devices according to the thin film vibrator. Moreover, the total input energy stored in PZT thin films is low, making it difficult to translate into high torques suitable for practical applications [19]. 

3D printing could fabricate piezoelectric elements; a piezoelectric-composite slurry was 3D printed using Mask-Image-Projection-based Stereolithography technology, and the printed ceramic exhibited an excellent piezoelectric constant and relative permittivity [20]. Simultaneously, piezoelectric elements could also be used to prepare a 3D microstructure for biological application, such as biosensors, cell-based assays, and microscale scaffolds of proteins [21]. Electroactive materials incorporated in 3D printing were introduced to 4D printing, making it possible to deliver electrical signals as well as actuating or sensing under external mechanical stimuli [22].

Many structures to improve the efficiency of energy conversion for piezoelectric devices were proposed [23], which combined piezoelectric materials and an auxetic design, with a negative Poisson’s ratio, for specific applications, such as the flexible membrane of a piezoelectric pulse sensor [24], cantilever beam energy harvester [25], and piezoelectric rain energy harvester [26], which demonstrated improved sensitivity output and the harvesting power. Further, Farhangdoust et al. proposed kirigami and auxetic topologies to design a metamaterial-based substrate for piezoelectric energy harvesters, which showed its strong potential for high-sensitive MEMS sensing applications [27]. In addition, Liu et al. introduced a multi-level surrogate model optimization scheme integrated with the optimal Latin hypercube design of experiments, maximizing energy conversion from human motion using a piezoelectric flex transducer [28]. Erturk et al. discussed the use of piezoelectric materials in conjunction with various nonlinear structures for frequency bandwidth enhancement in vibration energy harvesting [29]. Lu et al. reported a strategy for energy harvesting from the heart motion by using an ultra-flexible piezoelectric device based on PZT [30].

This paper proposes a method of preparing ring-type rotary traveling wave microscale piezoelectric vibrators based on printed PZT thick films without the use of a glue layer. In this study, the PZT thick film micro vibrator was simulated to optimize the elastic body structure to reduce the resonant frequency and obtain high vibration performance, where a modal analysis on micro vibrators using ANSYS finite element analysis (FEA) software as well as a transient dynamic analysis was conducted. Furthermore, an optimized micro vibrator was fabricated through printing a PZT thick film element on titanium bases directly using the electrohydrodynamic (E-Jet) printing technique, which was then combined via high temperature co-firing sintering to form micro piezoelectric vibrators. This process avoided the adhesion glue fabrication process, reduced the influences caused by the glue layer such as low conductivity, voltage drops, and isolating vibration typically occurring in the bulk piezoelectric vibrator fabrication technique. It also improved the piezoelectric element position accuracy corresponding to the bases, enhancing the performance of the piezoelectric vibrator. Consequently, vibration characteristics encompassing vibration amplitude, mode shape, and resonance frequency of the micro piezoelectric vibrator were analyzed. Then, the experimental results along with the simulation results were compared in order to verify the mode shape generation in micro piezoelectric vibrators. Finally, the dynamic driving performance of the piezoelectric vibrator was further tested by driving an annual rotor.

## 2. Experimental Details

### 2.1. Design of the PZT Thick Film Micro Vibrator 

**Figure 1:**
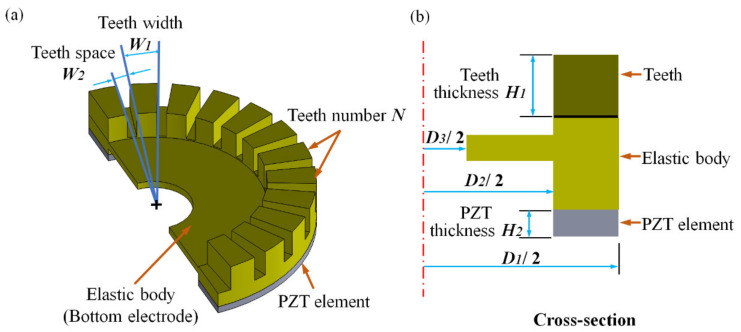
(**a**) PZT thick film micro vibrator structure and (**b**) cross-section of the micro piezoelectric vibrator.

The structure of the PZT thick film micro vibrator is shown in Figure 1a, which includes a ring shape PZT thick film element as well as an elastic body that serves as a metal base. The elastic body was made of titanium, which is a type of conductive material and can be used as a ground electrode. Therefore, there is no need to prepare an additional bottom electrode. The PZT thick film element, utilizing the converse piezoelectric effect, was capable of producing a vibration, and the elastic body with teeth evenly distributed on its working surface was used to amplify the bending vibration amplitude so as to form the elliptical motion. Moreover, the PZT thick film micro vibrator was ring shaped, which was able to produce a rotary traveling wave in the elastic body. As shown in Figure 1a,b, the outer diameter *D*_1_ of the elastic body of the piezoelectric vibrator was preliminary designed to be 4.3 mm, reducing the size of the piezoelectric vibrator and benefiting the maintenance of torque related to the outer diameter [31]. The thickness of the elastic body was determined to be 550 μm; the thickness of the PZT thick film element *H*_2_ was 35 μm; the diameter of the middle hole *D*_3_ for aligning and fixing the vibrator was 1100 μm; and the teeth thickness *H*_1_ was 250 μm. Other parameters such as the angle *W*_1_ of teeth width, the angle *W*_2_ teeth space, teeth number *N*, and inner diameter *D*_2_ of the elastic body were more important roles in designing PZT thick film micro vibrators, which were closely related to the resonance frequency, the vibration amplitude, and output performance of the micro vibrator directly. Hence, optimizing the dimension parameters of the elastic body via FEA using the ANSYS software is necessary. The ratios *D*_2_/*D*_1_ of the thick film micro piezoelectric vibrator were set as 0.4, 0.5, 0.6, 0.7, 0.8, and 0.9. Similarly, with the other size invariant, the teeth number *N* of the elastic body was 0, 10, 18, 20, 24, 30, 36, and 72. In addition, the different ratios *W*_2_/*W*_1_ of the elastic body were set as 1:5, 1:3, 1:2, 1:1, 2:1, 3:1, and 5:1. 

During the simulation process, the degree of freedom of the middle hole of the elastic body was set to zero, and the other boundaries were in a free state. As the parameter optimization process was based on FEA, it did not consider the influence of the adhesive layer, making the simulation process simple. Thick-film piezoelectric elements were printed on the substrate, and the d_33_ value of this kind pure PZT thick film was obtained from our previous work, which was around 140 pC/N [32]. Then, the d_31_ can be inferred from the relation described by C. Ayelas and L. Nicub [33]. The parameters of each material of the PZT film micro vibrator for FEA are shown in Table 1.

The working mode Bmn is a very important parameter for the ring-type piezoelectric vibrator; m and n indicate nodal circles and diameters, respectively, which are closely related to the resonant frequency and vibration amplitude of the piezoelectric vibrator. In terms of the micro piezoelectric vibrator having a diameter of a few millimeters, the natural resonant frequency is generally high. Here, m was usually set to 0 while n was set to 3, which was selected for the PZT thick film micro piezoelectric vibrator as the B03 mode maximized vertical displacement compared to higher-order modes, minimizing the required number of actuation regions. Subsequently, four electrodes were symmetrically designed on the ring-shaped PZT thick film element, which are used to excite the piezoelectric vibrator, and the distance between the two side electrodes was one quarter of the wavelength. Additionally, an isolated electrode was also designed to deliver feedback of the working state of the micro piezoelectric vibrator.

### 2.2. Preparation of the PZT Thick Film Micro Vibrator

The ring type PZT thick film element for the micro piezoelectric vibrator was produced using the E-Jet printing technique with a PZT composite slurry. The printing composite slurry used for making the PZT thick film element was prepared by thoroughly mixing the PZT powder and PZT sol. The details of the properties as well as the proportions of the composite slurry were described in previous studies [32,34]. Here, the slurry with a 1:1 ratio of PZT sol: PZT powder that was ball milled for 50 h was selected for the preparation of the printing material. The PZT composite slurry was of hard type with high Q_m_, which was appropriate for the piezoelectric element in applications requiring high power such as ultrasonic cleaning, ultrasonic motor, and ultrasonic polishing [35]. The material for making the elastic body was titanium, which had a matched acoustic impedance to PZT, reducing the energy loss during energy transfer [36]. Furthermore, its good elasticity, higher melting point, and low density satisfied the requirements of the fabricating process [37].

**Figure 2:**
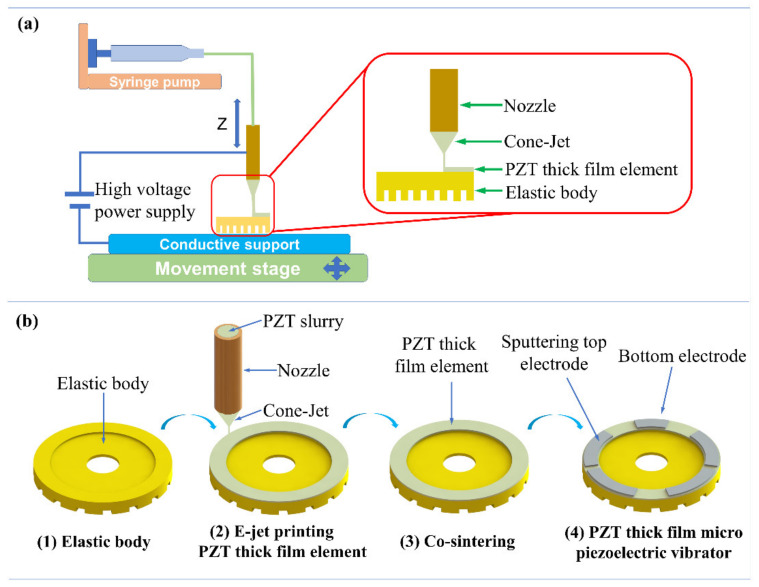
(**a**) Schematic diagram of E-Jet printing equipment and (**b**) preparation of the PZT thick film micro vibrator.

Figure 2a displays the E-Jet printing equipment, mainly consisting of a syringe pump (PHD ULTRA 70–3307, Harvard Apparatus, USA), a conductive support, a movement stage, and a high voltage power supply (DWP-P502-50ACF3, Tianjin Dongwen High Voltage Power Supply Co., Ltd., Tianjin, China). The nozzle, with the outer/inner diameter of 0.7/0.2 mm, was connected to the high voltage power supply, which was used to provide an electric field between the nozzle and the conductive support. A conductive support made of aluminum, as a ground electrode, was placed on the movement stage and could move along the preset graphical trajectory. The PZT slurry was pushed to the outlet of the nozzle due to the hydrodynamic force provided by the syringe pump. The electric field causes mobile ions in the PZT composite slurry to accumulate at the liquid surface, resulting in a meniscus at the nozzle end to deform into a Taylor cone and a fine jet; subsequently, the PZT thick film element was produced on the elastic body using the E-Jet printing process.

The fabricating process is shown in Figure 2b. The elastic body was fixed on an aluminum conductive support. Then, the thick film element was prepared by using printing parameters of a flow rate of 1.67 × 10^−11^ m^3^·s^−1^, a voltage of 0.12 kV, a working distance of 0.45 mm, and a travelling speed of 11 mm/s. Moreover, for each layer of printed PZT film, it was necessary to heat the PZT thick film micro vibrator in order to remove the residual organic solvents in the PZT film. Additionally, the heating procedure was comprised of two parts: 200 °C front heating for 1 min and 350 °C back heating for 1 min. Subsequently, the elastic body with the printed PZT thick film element was co-sintered in order to obtain a completed perovskite structure at 700 °C for 20 min [21]. During the process, the PZT thick film element and Ti elastic body combined with each other via high temperature annealing, ensuring the bonding force during vibration. The PZT thick film element and the elastic body were integrated together tightly without the use of glue, which can increase the vibration amplitude and reduce the driving voltage. Ti/Pt electrodes with a thickness of 50/200 nm were prepared using magnetron sputtering on the PZT thick film element so as to form the PZT thick film micro vibrator. Finally, the PZT thick film element with five electrodes was poled at an electric field of 10 V/µm and a temperature of 200 °C for 30 min to improve the piezoelectric properties, and to ensure the polarization direction of the adjacent electrodes opposite in forming the travelling waves. For differently sized PZT thick film devices and batch manufacturing, all that is needed is to change the movement platform parameters and keep other printing parameters constant. Therefore, E-jet printing has a certain potential on scalability and batch production possibilities.

### 2.3. Vibration Measurement for the PZT Thick Film Micro Vibrator

**Figure 3:**
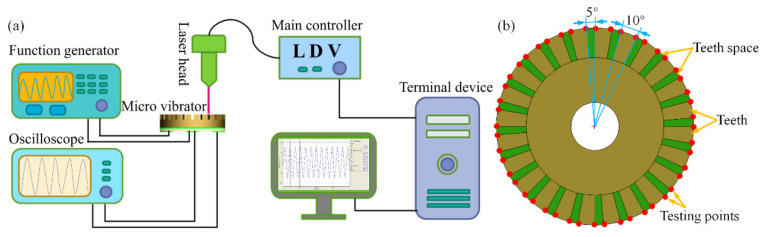
(**a**) Diagram of amplitude measuring experimental system; (**b**) Distribution of testing points on the teeth of the ring-type micro vibrator.

The amplitude distribution on the teeth surface of the PZT thick film micro vibrator was tested using the amplitude measuring experimental system. As shown in Figure 3a, the system included a laser head and main controller terminal device in conjunction with a function generator (33521B, Keysight technologies, Santa Rosa, CA, USA), digital oscilloscope (TBS1102, Tektronix, Beaverton, OR, USA), and single-point laser Doppler vibrometer (LDV) system (OFV-534/VDD, Polytec, Waldbronn, Germany) [38]. The function generator provided a sinusoidal voltage at a specific frequency as the power supply. The amplitudes of any position of the piezoelectric vibrator were measured by the laser head by adjusting the displacement. The main controller then quickly calculated parameters such as amplitude and velocity of the test point, after which the processed data were displayed on the terminal device. The oscilloscope was used to detect the feedback signal of the isolated electrode of the PZT thick film micro piezoelectric vibrator, reflecting the vibration of the micro piezoelectric vibrator. The displacement tests were carried out on the vibration isolation platform in order to eliminate the influence of random vibrations from the external environment. Figure 3b depicts a top view image of the ring-type micro vibrator with its teeth up. Here, the left and right points of the vibrator’s outermost ring circle of each tooth serve as the test points (red). A total of 48 points (24 teeth × 2) were distributed in the circular direction evenly and measured in sequence. Furthermore, the angle of one tooth width in the circumferential direction was 10°, while the angle of tooth space width was 5°. 

## 3. Results and Discussion

### 3.1. Optimization of PZT Thick Film Micro Vibrator

**Figure 4:**
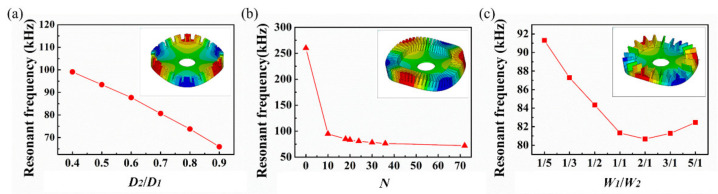
(**a**) Ratio of inner to outer diameters *D*_2_/*D*_1_ of the piezoelectric vibrator versus resonant frequency; (**b**) Teeth number *N* of the piezoelectric vibrator versus resonant frequency; (**c**) Width ratio of tooth to tooth space *W*_1_/*W*_2_ versus resonance frequency.

The relationship between the ratio of inner to outer diameters (*D*_2_/*D*_1_) and the resonant frequency is shown in Figure 4a. The corresponding resonant frequencies declined with the increase of the ratio of *D*_2_/*D*_1_ in the B03 modes. It can be seen that a higher ratio will reduce the resonant frequency and increase the vibrational amplitude of the vibrator. However, it also results in the smaller utilization area of the piezoelectric element, which will reduce the driving performance of the PZT thick film micro vibrator. Subsequently, the ratio (*D*_2_/*D*_1_) of 0.7 was chosen in this vibrator, and then the inner diameter of the elastic body was 3 mm. Figure 4b depicts the teeth number *N* of elastic body as 0, and the micro piezoelectric vibrator had a particularly high resonance frequency of around 260 kHz in B03. However, the resonance frequency went sharply downward to 98 kHz at a point of 10 teeth, which indicated that the teeth structure had a significant influence on the resonant frequency of the thick film micro piezoelectric vibrator. Moreover, due to the increase in teeth number, the resonance frequency decreased; however, when the number of teeth was higher than 20, the teeth number’s influence on the resonant frequency was not obviously apparent. Usually, the teeth number on the elastic body is generally a multiple of traveling waves generated in the piezoelectric vibrator; therefore, 24 teeth were designed on the elastic body. The relationship between resonant frequency and width ratio of the tooth to tooth space *W*_1_/*W*_2_ is shown in Figure 4c. Here, the resonance frequency initially dropped but rose with the increasing ratio in B03, where the frequency was at its minimum at ratio 2. The piezoelectric vibrator resonance frequency gradually increased as the ratio rose. When the ratio was greater than 2, the equivalent thickness and rigidity of the elastic body increased. Therefore, the width ratio of the tooth and teeth space *W*_1_/*W*_2_ was selected to be 2 with the minimum resonant frequency. With the designed teeth thickness *H*_1_ of 250 μm as well as the thickness of the PZT element *H*_2_ of 35 μm, the size parameters of the PZT thick film micro vibrator are shown in Table 2, which were optimized according to the result of FEA.

### 3.2. Modal and Transient Analysis of the PZT Thick Film Micro Vibrator

Modal analysis can be used to obtain the working mode resonant frequency and modal shape of the PZT thick film micro vibrator. The optimized parameters of the PZT thick film micro vibrator are shown in Table 2, and the coupling simulation model was established with the APDL simulation script. Obviously, the simulation process did not need to consider the influence of the glue layer, which simplified the simulation process compared to a bulk manufacturing process considering glue [12].

**Figure 5:**
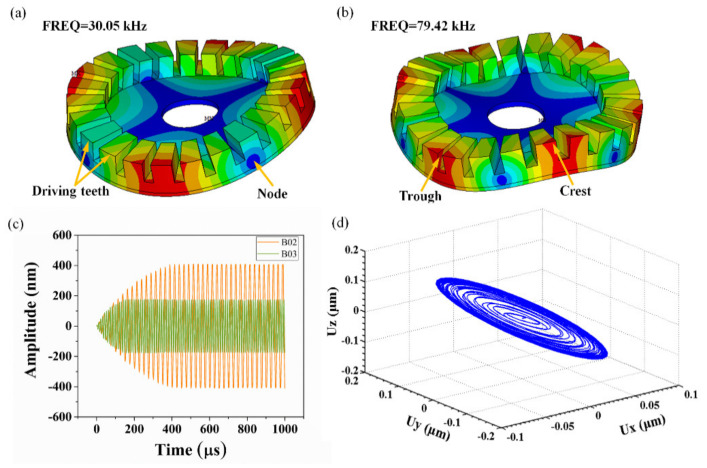
(**a**,**b**) Mode shapes simulated at resonant frequencies of 30.05 kHz and 79.42 kHz; (**c**) Amplitude-time curve in transient dynamic analysis at vertical Z direction; (**d**) Ellipse motion trajectory of one surface particle in three-dimensional space.

A supersonic frequency mode above 20 kHz analysis was used as the piezoelectric vibrator worked in the supersonic frequency band [39]. According to the structure design and fundamental piezoelectric vibrator operations, the primary interest modes were B02 and B03, which consisted of 2 and 3 standing waves, respectively. Additionally, the modal analysis demonstrated that the natural frequencies of the micro piezoelectric vibrator were 30.05 kHz and 79.42 kHz with corresponding modes of B02 and B03, respectively. Figure 5a shows that B02 is comprised of two complete standing waves, where each standing wave contains 2 nodes and 12 driving teeth. Similarly, as shown in Figure 5b, B03 consisted of 3 standing waves with 6 nodes, where each standing wave contained one crest and one trough. Figure 5c depicts the steady-state amplitude of B02 and B03 modes of the micro piezoelectric vibrator obtained by the transient dynamic simulation at a specific frequency excitation. Using the full method of transient dynamics simulation, the parameters of excitation voltage amplitude was 10 V, and the excitation frequencies were 30.05 and 79.42 kHz, respectively. The amplitude of the testing point on the surface of the micro piezoelectric vibrator became steady with time in the Z direction. The B02 mode amplitude of the micro piezoelectric vibrator tended to become stable after 0.4 µs, and the B03 mode was stable after 0.2 µs, where the maximum stable amplitude was 417 nm in B02, and 184 nm in B03. As shown in Figure 5d, the displacement data of one surface particle of the micro piezoelectric vibrator in the three-dimensional space coordinate system were acquired. The movement track in the three-dimensional space was an oblique ellipse, demonstrating that the trajectory of the piezoelectric vibrator surface particles obtained by the transient dynamics analysis were consistent with the theoretical results [40]. 

### 3.3. Vibration Characterization of PZT Thick Film Micro Vibrator 

**Figure 6:**
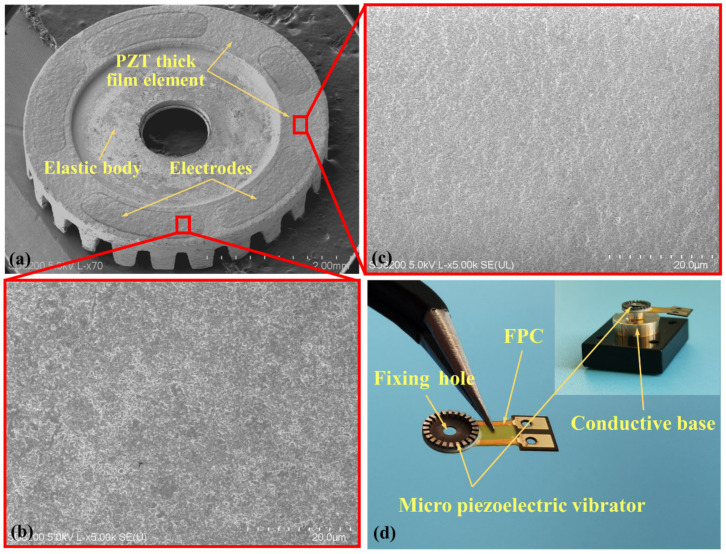
(**a**) PZT thick film micro vibrator, (**b**) micro morphology of printed thick films, (**c**) micro morphology of sputtered electrode, and (**d**) structure of assembled micro piezoelectric vibrator.

Figure 6a shows the PZT thick film micro vibrator with a thickness of less than 600 µm, which was composed of an elastic body, a PZT thick film element, and electrodes. Moreover, the diameter of the middle hole (1100 µm) enabled the alignment and fixing of the micro vibrator. The PZT thick film element served as the core component of the piezoelectric micro vibrator, and the surface of the PZT thick film was smooth and defect-free. Such attributes are important in providing reliable mechanical and electrical properties and generating a stable driving force in piezoelectric vibrators. By co-sintering the PZT thick film element and elastic body, a whole vibrator was formed without interface cracks, and this improved the bonding force between them, which is beneficial to the direct vibration transmission as well as the performance of the micro piezoelectric vibrator. Two pairs of electrodes along with a single electrode were symmetrically distributed on the PZT thick film element with a width of 450 µm. A pair of electrodes on one side acted as one group, where the first group of electrodes was used to excite the cosine mode, while the sine mode was excited by another pair of electrodes so as to produce a traveling wave. Figure 6b shows the printed thick film micro morphology, which is smooth without obvious physical damage. The grain size of PZT thick film is regular and connected together, which is beneficial to the electrical properties of piezoelectric thick films. Figure 6c depicts the sputtered electrode layer; the electrode layer is relatively uniform and well connected, ensuring that the vibrator can be applied to excitation voltage correctly according to the partition area. Figure 6d demonstrates the assembled PZT thick film micro vibrator, which was fixed on the conductive base with PFC connecting to the top electrodes.

**Figure 7:**
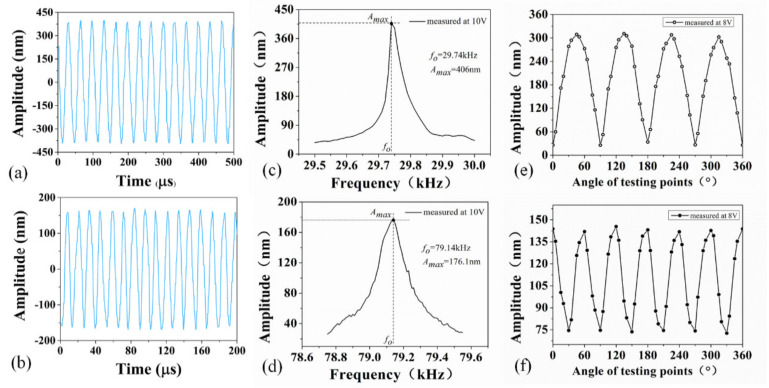
(**a**,**b**) Steady-state amplitudes curves of the B02 and B03 modes; (**c**,**d**) Amplitude-frequency curves of the B02 and B03 modes; (**e**,**f**) Amplitude distribution of micro piezoelectric vibrator in circumferential direction of the B02 and B03 modes.

Figure 7a,b show the steady-state amplitudes of the B02 and B03 modes. These two figures depicted that the normal vibration displacement curves were also approximately sinusoidal with the periodic changes. Correspondingly, the minimum and maximum amplitudes were noted to change slightly, possessing good symmetry and consistency. The results demonstrated that the PZT thick film micro vibrator is stable in vibration and has good driving ability. According to Figure 7c, the excitation voltage was 10 V, and the driving frequency varied from 29.50 kHz to 30.00 kHz. The amplitude initially increased, and then decreased, and the largest amplitude of the test points was 406 nm, illustrating that the corresponding resonant frequency of the micro piezoelectric vibrator was 29.74 kHz. The vibration amplitude of the thick film piezoelectric vibrator is large, which is comparable with the piezoelectric bulk vibrator; however, the driving voltage of the thick film piezoelectric vibrator was much smaller [41,42]. One main reason is that the transmission is more direct from the piezoelectric element to the elastic body without the glue layer. Since the glue layer isolates and absorbs the strain output of the piezoelectric element, even the rigidity constraint of the glue layer reduces the deformation of the piezoelectric element, resulting in a relatively small amplitude of the micro piezoelectric vibrator. Similarly, Figure 7d shows that the amplitude curve fluctuated from 78.75 kHz to 79.55 kHz. The maximum amplitude of the test point was 176.1 nm, and the corresponding resonant frequency of the test point was 79.14 kHz, indicating that the resonant frequency of B03 was 79.14 kHz. Figure 7e depicts the amplitude distribution on the micro piezoelectric vibrator in the circumferential direction measured by a single-point LDV at a frequency of 29.74 kHz. The amplitudes of the 48 test points were measured in sequence in the circumferential direction with an input voltage of 8 V. Here, four crests and four troughs were present on the amplitude break line with each cycle having 12 test points, indicating that the micro piezoelectric vibrator formed two complete waveforms with four nodes, where each waveform included one crest and one trough. The observed mode shape results are consistent with the simulation results of the B02 mode. As shown in Figure 7f, at a frequency of 79.14 kHz, the amplitudes of 48 test points were arranged sequentially due to the same reason, indicating that three complete waveforms with a maximum amplitude of 142 nm were formed at an excitation voltage of 8 V. Similar to the simulation results, the amplitude break line had three complete waveforms comprised of six nodes, each of which contained eight points.

The testing results measured by LDV were compared to that of the simulation, as shown in Table 3. With regard to the resonant frequency, the B02 mode’s simulation frequency was 30.05 kHz, which was slightly higher than that measured by LDV with a relative error of 1.03%. Similarly, the B03 vibration modes that had a difference of 0.28 kHz were very close to the testing value, where the difference between the simulation results and the test results was less than 0.35%. In view of the maximum amplitude, the simulation result of the B03 modal was 184 nm, while the actual test amplitude was 176 nm with an error of 4.35%, and the error of the B02 modal was 2.64%. All results had errors less than 5%, which verified the accuracy of the simulated results in this study, effectively optimized the structure of the elastic body, and realized the low resonance frequency and large amplitude of the PZT thick film vibrator. The results also further proved that the micro piezoelectric vibrator formed the B02 and B03 vibration modes at the corresponding frequencies successfully, and it has a significant guiding on the design, manufacturing, and subsequent vibration testing of the PZT thick-film micro vibrator. The error of the simulation result was smaller than that of the simulation value when considering the influence of the adhesive layer [43] because the preparation of the PZT thick film micro piezoelectric vibrator was simplified, in which the piezoelectric element was directly printed onto the elastic body. Furthermore, the simulation model was also simpler, avoiding uncertain factors caused by the glue layer. 

**Figure 8:**
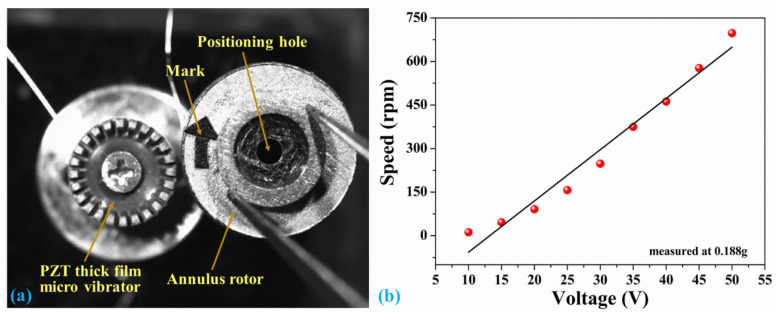
(**a**) PZT thick film micro vibrator and rotor and (**b**) the curve between excitation voltage and rotating speed.

The dynamic driving performance of the piezoelectric vibrator was tested, after forming the correct vibrational modes. An annulus rotor made by stainless steel was driven by the micro vibrator, and the rotation speed was further tested using a high-speed camera (Mikrotron, Germany). Two sinusoidal excitation voltages with a phase difference of ±90 degrees were applied on the micro vibrator, and then the vibrator forms traveling waves to drive the rotor bidirectional rotation. The micro vibrator and rotor were shown in Figure 8a; the annular rotor with a weight of 0.188 g was directly placed on the micro vibrator, which provides a preloading force of 1.88 mN on the micro vibrator. Then, the rotor was driven by the friction between the vibrator and the rotor. The middle hole in the rotor is a positioning hole for the rotation process, and the arrow mark shows the rotational direction. Figure 8b shows the relationship between the excitation voltage and rotating speed with the preloading force of 1.88 mN at the B03 mode. The speed increased with increasing excitation voltage at the resonant frequency of 79.14 kHz. The maximum rotating speed of the rotor driven by the vibrator was up to 681 rpm with the excitation voltage of 50 V, and the sinusoidal excitation voltage range was from 10 to 50 V. Moreover, linear relationships between the rotating speed and excitation voltage are observed, which indicated high operation control of this PZT thick film micro vibrator.

## 4. Conclusions

In this paper, a novel technology used for the preparation of a rotary traveling wave micro piezoelectric vibrator based on E-Jet printing PZT thick film was proposed. The PZT thick film element was printed directly onto the elastic body and was combined by co-sintering at 720 °C for 20 min. This method eliminated the glue layer usually used in the bulk PZT fabrication process, and then the effects caused by adhesive glue of voltage drops; low conductivity resulted, and isolating and absorbing amplitudes were avoided. The simulation model of the PZT thick film micro vibrator was established in order to optimize the structure parameters of the elastic body, and the optimized parameters of the micro piezoelectric vibrator encompassed having an external diameter of 4.3 mm, an internal diameter of 3 mm, a teeth number of 24, a width ratio of teeth to teeth space of 2:1, a thickness of 585 µm, and a PZT element thickness of 35 µm. This optimized structure can reduce the working resonant frequency and increase the vibration amplitude of the piezoelectric micro piezoelectric vibrator with the same excitation voltage. The simulation of resonant frequencies were 30.05 kHz and 79.42 kHz, while the amplitude simulation value of B03 was 184 nm. The theoretical elliptical motion track of the surface particle of the micro piezoelectric vibrator was also formed. By utilizing LDV testing, the B02 mode resonant frequency of the micro piezoelectric vibrator was 29.74 kHz with a large amplitude of 406 nm, while that of the B03 mode was 176 nm with a resonance frequency of 79.14 kHz. This large amplitude is due to the problems involving voltage drops, and isolating and absorbing amplitudes caused by glue layer were avoided. The error between the simulation and test result in the B03 modal is only 0.35%, which proves the accuracy of the FEA simulation method. In addition, the simulation process is much simpler for this structure without the glue interface layer. The FEA effectively optimized the structure of the elastic body, and realized the low resonance frequency and large amplitude of the PZT thick film vibrator. The micro vibrators realized bidirectional rotation of a rotor with a maximum speed of 681 rpm, and which also indicated high operation control of this PZT thick film micro vibrator due to the linear relationships between the rotating speed and excitation voltage. This study provided a promising method of developing unique PZT thick film micro piezoelectric vibrators by printing a PZT thick film element onto an elastic body by utilizing E-Jet printing. Such products have a large vibrating amplitude and low excitation voltage, which may be applied in MEMS ultrasonic devices such as rotary type piezoelectric motors or piezoelectric polishing.

## Figures and Tables

**Table 1:** Material constants for simulation (*t* = 20 °C).

Material	PZT	Titanium Elastic Body
Elastic stiffness matrix (×10^9^ N/m^2^)	$\left(\begin{array}{cccccc} 168 & 110 & 99 & 0 & 0 & 0\\ 110 & 168 & 99 & 0 & 0 & 0\\ 99 & 99 & 123 & 0 & 0 & 0\\ 0 & 0 & 0 & 29 & 0 & 0\\ 0 & 0 & 0 & 0 & 30 & 0\\ 0 & 0 & 0 & 0 & 0 & 30 \end{array}\right)$	110
Piezoelectric stress matrix (C/m^2^)	$\left(\begin{array}{ccc} 0 & 0 & -3.28\\ 0 & 0 & -3.28\\ 0 & 0 & 5.07\\ 0 & 0 & 0\\ 0 & 4.68 & 0\\ 4.68 & 0 & 0 \end{array}\right)$	-
Relative dielectric matrix	$\left(\begin{array}{ccc} 828 & 0 & 0\\ 0 & 828 & 0\\ 0 & 0 & 700 \end{array}\right)$	-
Poisson’s ratio	-	0.34
Density (kg/m^3^)	7500	4500
Curie temperature, *T*_C_ (°C)	300	-

**Table 2:** Optimized structural dimensions of micro piezoelectric vibrator (mm).

Parameters	*D*_1_	*D*_2_	*W*_1_ */W*_2_	*N*	*H*_1_	*H*_2_
Value	4.3	3.3	2:1	24	0.25	0.035

**Table 3:** Simulation and test results for the PZT thick film micro vibrator.

Vibration Mode	FEA Frequency (kHz)	LDV Frequency (kHz)	Frequency Percent Error (%)	FEA Amplitude (nm)	LDV Amplitude (nm)	Amplitude Percent Error (%)
B02	30.05	29.74	1.03	417	406	2.64
B03	79.42	79.14	0.35	184	176	4.35
